# Induration or erythema diameter not less than 5 mm as results of recombinant fusion protein ESAT6-CFP10 skin test for detecting *M. tuberculosis* infection

**DOI:** 10.1186/s12879-020-05413-9

**Published:** 2020-09-18

**Authors:** Hui Zhang, Ling Wang, Feng Li, Shuihua Lu, Jielai Xia

**Affiliations:** 1 Department of Health Statistics, The Fourth Military Medical University, No. 169 Changle West Road, Xi’an, Shaanxi China; 2 Department of Tuberculosis Control and Prevention, Xi’an Center for Disease Control and Prevention, Xi’an, Shaanxi China; 3 Shanghai Public Health Clinical Center, Fudan University, Shanghai, China

**Keywords:** *Mycobacterium tuberculosis*, Skin test, ESAT6-CFP10, Diameter

## Abstract

**Background:**

Recombinant fusion protein ESAT6-CFP10 (EC) is a newly developed skin test reagent for detecting *Mycobacterium tuberculosis* (*M. tuberculosis*) infection. In this study, we evaluated whether induration and erythema could be used as diagnostic indicators for EC skin test to detect *M. tuberculosis* infection.

**Methods:**

A total of 743 tuberculosis patients and 1514 healthy volunteers underwent an EC skin test. The diameters of induration and erythema were measured with Vernier caliper, 24 h, 48 h, and 72 h after skin testing. Related indicators of EC reagent diagnostic test were tested, and the diagnostic effects of the four diagnostic indicators for EC skin test were compared.

**Results:**

The sensitivity of induration / erythema measurement was lower at 24 h after EC skin test than at 48 h or 72 h (*P*<0.01). There was no difference in consistency (*P* = 0.16) between induration with clinical diagnosis, and erythema with clinical diagnosis at 48 h (88.88 and 90.16%, Kappa value was 0.75 and 0.78, respectively). In patients, the sensitivity of erythema measurement was higher than induration measurement (*P*<0.01). In healthy volunteers, the specificity of erythema measurement was lower than induration at 24 h after skin test, but there was no difference at 48 h after skin test (*P* = 0.22). In BCG vaccination volunteers, the specificity of induration and erythema were higher than 90%. In addition, there was a high consistency of induration and erythema. When induration or erythema was used as a positive diagnostic indicator, the sensitivity of the EC skin test was improved, and was no different from the other three indicators in terms of specificity and consistency with clinical diagnosis.

**Conclusions:**

Induration or erythema diameter not less than 5 mm could be used as a diagnostic indicator for detecting *M. tuberculosis* infection.

**Trial registration:**

Phase III clinical trial of recombinant *Mycobacterium tuberculosis* ESAT6-CFP10 allergen; CTR20150695; registered in December 16, 2015.

## Background

Tuberculosis (TB) remains a leading cause of morbidity and mortality, and a major public health problem worldwide [1]. World Health Organization (WHO) has estimated that 10.4 million new cases and 1.8 million deaths from TB occur every year [2, 3]. Approximately, one-third of the world’s population is infected with *Mycobacterium tuberculosis* (*M. tuberculosis*), and about 1.7 billion people have latent tuberculosis infection (LTBI) [4, 5], especially in developing countries [1]. China is one of the countries with a high burden of TB and LTBI. Although the prevalence of TB is gradually decreasing [6], it still remains an important threat due to its high infectiousness. The Chinese government is making strenuous efforts to prevent further spread of infection and provide rapid detection and treatment facilities through national support and project grants [7].

Currently, the most common method used to detect LTBI implies testing the hosts’ immune response to *M. tuberculosis* derived antigens [8, 9]. Tuberculin pure protein derivative (PPD) skin test and Interferon-γ (IFN-γ) release assays (IGRAs) are commonly used for detecting *M. tuberculosis* infection [10–12], but the tuberculin skin test (TST) has a high false positive rate [13, 14]. IGRAs is an immunodiagnostic test that measures effector T cell-mediated IFN-γ response to *M. tuberculosis*-specific antigens. Both 6 kDa early secretory antigenic target (ESAT6) and 10 kDa culture filtrate protein (CFP10) are co-transcribed and specifically secreted by *M. tuberculosis* [15, 16], which is absent in most non-tuberculous mycobacteria (NTM) and Bacillus Calmette-Guérin (BCG) [17–20]. IGRAs can limit the false positive reaction [21, 22] and overcome the operational drawbacks of TST [23]. However, the detection reagents used for IGRAs are expensive, and the operation process is difficult to be automated [24]; thus, IGRAs are not suitable for use in remote areas and wide-range screening.

According to the characteristics of *M. tuberculosis-*specific antigens, China has recently developed a recombinant fusion protein ESAT6-CFP10 (EC) as a new skin test reagent, which was expected to have high sensitivity and specificity. In this study, we conducted large sample research to clarify whether induration and erythema diameter could be used as diagnostic indicators for EC skin test to detect *M. tuberculosis* infection.

## Methods

### Clinical trial units

The study on TB patients was completed by Shanghai Public Health Clinical Center, Tianjin Haihe Hospital, Wuhan Tuberculosis Prevention and Treatment Center, Beijing Chest Hospital, First Affiliated Hospital of Chongqing Medical University, Wuxi Infectious Disease Hospital, Fuzhou Pulmonary Hospital, Zhenjiang Third People’s Hospital, Anhui Provincial hospitals and Shenzhen Third People’s Hospital; Shanghai Public Health Clinical Center was the main responsible unit. The research was performed from December 17, 2015 to March 02, 2018. Jiangsu Provincial Center for Disease Control and Prevention completed the research on healthy volunteers from February 27, 2016 to June 18, 2016.

The Human Research Ethics Committee granted the research protocol. All participants read and signed the informed consent.

### Inclusion and exclusion criteria

Healthy volunteers aged 18 to 65 were recruited and included in this study if they met the following criteria: (a) no history or family history of TB; (b) no X-ray chest abnormality; (c) normal physical condition and temperature. Healthy volunteers were excluded if they (a) had serious diseases such as advanced cancer, acute exacerbation of the chronic obstructive pulmonary disease, acute or progressive liver disease, acute or progressive kidney disease or congestive heart failure; (b) had a mental illness; (c) had known or suspicious immune function impairment or abnormality; (d) had an acute febrile illness and infectious disease; (e) were currently participating in other drug clinical trials or have participated in any other new drug clinical trials within 3 months before the testing; (f) had a history of drug allergy; (g) were pregnant or lactating; (h) had a clear history of hypertension; (i) had other reasons that investigators believed might affect the evaluation of the test.

TB patients’ inclusion criteria were: patients 18 to 65 years old who were definitively diagnosed according to the criteria for pulmonary TB of the Ministry of Health of China (WS 288–2008, supplementary). TB patients were excluded if they met the (a) (b) (e) (f) (g) (i) six items in the health volunteers’ exclusion criteria.

Finally, 743 TB patients and 1514 healthy volunteers were enrolled in this study.

### Skin test reagent

ESAT6-CFP10 included the following: a recombinant fusion protein of ESAT6 and CFP10, which was manufactured by Anhui Longcom Biologic Pharmacy Co. Ltd. This reagent was supplied as a liquid formulation (0.3 ml/bottle), consisting of ESAT6-CFP10 antigen (50 U/10μg/ml), phosphate-buffered saline (PBS, 1.0 mmol/liter), phenol (3%) and Tween 80 (0.0005%).

### Skin test operation

Before starting the skin test, skin test reagent, rescue medicine and rescue equipment were prepared. Each numbered skin test reagent was only be used by one subject and could not be mixed with others. The authorized medical staff checked the drug number/subject number of each subject, and then checked the expiration date, bottle wall, and drug contents.

The skin was sterilized with ethanol in the first 1/3 of the subjects’ forearm volar side without scars or lesions. Then, 0.1 ml skin test reagent was inhaled into a 1 ml disposable syringe, the scale and the tip hole slope were consistently upward. The medical staff’s left thumb tightened the skin of the subject where the injection was required, and the right hand hold the syringe, which penetrated into the skin at 5° ~ 10°from the skin. The reagent was injected slowly; the white rounded ridge was visible, the pores were revealed, and the boundary was clear after the injection. After the injection was completed, the needle was pulled out while rotating 90 degrees. Subjects were instructed not to get wet or rub the injection site for 72 h. Medical staff was required to change gloves for each patient to avoid contamination.

### Outcome and measurement

Previous literature reports and small sample clinical trial suggested that erythema can be used as a specific response to the skin test. In this study, outcome measures were induration and erythema diameters in every subject. All patients were observed at 24 h, 48 h, and 72 h after a skin test, and healthy volunteers were observed at 24 h and 48 h after skin test. Firstly, the medical staff analyzed the induration (touching the skin with fingers) and observed the erythema outer ring to determine edges of induration and erythema. The upper and lower edges (longitudinal diameter), left and right edges (transverse diameter) of the induration were marked by a short line with a black pen. The boundary of the erythema was marked by a short line with a red pen. The longitudinal and transverse diameters of induration and erythema were measured with Vernier caliper. Two medical staff measured and read the size of the skin test response to ensure the reading error between the two people controlled within ±0.2 mm. The averages of the longitudinal and transverse diameters was the sizes of induration and erythema diameter. A previous clinical trial showed that the area under the ROC curve was the largest when the cutoff value of the diameter of induration and erythema was 5 mm. Therefore, in this study, the diameter of induration and erythema of 5 mm was the positive criterion for the EC skin test response. This clinical trial expanded the sample size for further verification. So, the results of the EC skin test were considered positive when the diameter of induration or erythema was ≥5 mm, and strongly positive if a local blister, necrosis, or lymphangitis were observed.

### Statistical analysis

Statistical analysis was conducted using SAS 9.3 software. The diameters of induration and erythema after EC skin test in all subjects were described with mean ± SD, number, median, minimum and maximum. Sensitivity, Specificity, Positive predict value (PV_+_), Negative predict value (PV_−_), Youden index (YI), consistency, and Kappa value (95% confidence interval) were used to compare the consistency of induration / erythema and clinical diagnostic results. Positive coincidence rate (CR_+_), Negative coincidence rate (CR_−_), consistency, and Kappa value (95% CI) were carried out to compare the consistency of induration and erythema results. A Chi-square test was applied to compare the rates in different groups. *P* <  0.05 was considered to be statistically significant.

## Results

### Characteristics of participants

In total, 743 TB patients were enrolled in the study; they were 38.77 ± 14.11 years old, 513 subjects (69.04%) were male, and 118 subjects had a history or family history of TB. There were 1514 healthy volunteers, who were 45.43 ± 9.57 years old, and 398 subjects (26.29%) were male. Among them, 777 volunteers had BCG vaccination scars (were vaccinated during infancy, which was a basic health policy in China). BCG is very effective in preventing severe tuberculosis in childhood, but is less effective in adults [25–27]. Therefore, healthy adults vaccinated with BCG during infancy were considered as a generally healthy population without BCG protection. In order to evaluate the specificity of the EC skin test in the BCG vaccination population, 315 healthy volunteers were selected for BCG vaccination according to the purpose of the clinical trial. The other characteristics are shown in Table 1.

**Table 1:** Characteristics of enrolled TB patients and healthy volunteers

	TB patients (*N* = 743)	Healthy volunteers (*N* = 1514)
Age (y)	38.77 ± 14.11	45.43 ± 9.57
Height (cm)	167.93 ± 7.65	161.42 ± 7.19
Weight (kg)	57.48 ± 10.49	64.14 ± 10.63
Male, n (%)	513 (69.04)	398 (26.29)
Residence, town n (%)	487 (65.55)	–
Occupations	743	–
Staff, n (%)	249 (33.51)	–
Student, n (%)	52 (7.00)	–
Farmer, n (%)	94 (12.65)	–
Others, n (%)	348 (46.84)	–
Han nationality, n (%)	718 (96.64)	1507 (99.54)
Systolic blood pressure (mmHg)	117.59 ± 13.61	130.82 ± 16.88
Diastolic blood pressure (mmHg)	74.44 ± 9.38	79.48 ± 10.05
Heart rate (times/min)	83.75 ± 11.80	–
Breathe (times/min)	19.13 ± 1.73	–
Temperature (°C)	36.64 ± 0.48	35.98 ± 0.41
BCG vaccination, n (%)	–	315 (20.81)
Disease history within past 6 months, n (%)	213(28.67)	328 (21.66)
Combined medication history, n (%)	604(81.29)	272 (17.97)
Allergic history, n (%)	47(6.33)	–
Non-TB infectious history, n (%)	18(2.42)	–
Other disease history, n (%)	25(3.36)	–
History and family history of TB, n (%)	118(15.88)	–
Types of tuberculosis	743	–
Bacteriologically positive tuberculosis, n (%)	394 (53.03)	–
Bacteriologically negative tuberculosis, n (%)	282 (37.95)	–
Extrapulmonary tuberculosis, n (%)	67 (9.02)	–

### The diameters of induration and erythema after EC skin test

**Fig. 1:**
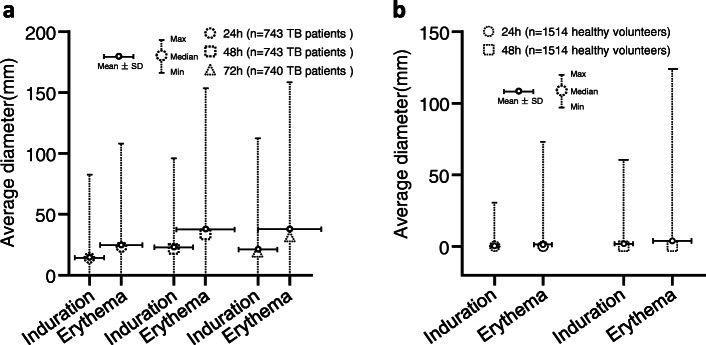
The diameters of induration and erythema after EC skin test. **a** The diameters of induration and erythema in TB patients. **b** The diameters of induration and erythema in healthy volunteers

In TB patients, the average diameter of induration was 14.23 mm, 22.68 mm, and 21.00 mm at 24 h, 48 h and 72 h after EC skin test, while the maximum diameter was 82.50 mm, 96.00 mm and 112.50 mm, respectively. Moreover, the average diameter of erythema was 24.72 mm, 37.55 mm, and 37.64 mm at 24 h, 48 h, and 72 h after EC skin test, while the maximum diameter was 108.00 mm, 153.50 mm and 158.50 mm, respectively (Fig. 1a).

In healthy volunteers, the average diameter of induration was 0.36 mm and 1.81 mm at 24 h and 48 h after EC skin test, while the maximum diameter was 30.50 mm and 60.50 mm respectively; the average diameter of erythema was 1.23 mm and 3.86 mm at 24 h and 48 h after EC skin test, and the maximum diameter was 73.00 mm and 124.00 mm, respectively (Fig. 1b).

### The diagnostic value of induration and erythema after EC skin test

The sensitivity of induration measurement was 72.14, 84.25, and 83.78% at 24 h, 48 h, and 72 h after EC skin test, respectively; the sensitivity was the lowest at 24 h (χ^2^ = 43.80, *P* < 0.01), while there was no statistical difference between the sensitivity of 48 h and 72 h (χ^2^ = 0.06, *P* = 0.81). The specificity of induration measurement was 97.49 and 91.15% at 24 h and 48 h after EC skin test, respectively; the specificity was lower at 48 h (χ^2^ = 56.81, *P* < 0.01). The Kappa value was 0.75 at 48 h. The consistency of 24 h and 48 h after the skin test between induration and clinical diagnosis was close to 90% (χ^2^ = 0.08, *P* = 0.78). The remaining results are shown in Table 2.

**Table 2:** The diagnostic evaluation of induration and erythema after EC skin test

Diagnostic index	Different times			χ^2^	*P*
	24 h	48 h	72 h		
Induration results					
Sensitivity	72.14%^a^	84.25%	83.78%	43.80	< 0.01
Specificity	97.49%^a^	91.15%	–	56.81	< 0.01
PV_+_	93.38%^a^	82.37%	–	35.30	< 0.01
PV_−_	87.70%^a^	92.18%	–	17.41	< 0.01
Consistency	89.14%^a^	88.88%	–	0.08	0.78
YI	69.63%	75.40%	–		
Kappa (95% CI)	0.74 (0.71, 0.77)	0.75 (0.72, 0.78)	–		
Erythema results					
Sensitivity	85.33%^a^	90.85%	91.08%	16.18	< 0.01
Specificity	95.38%^a^	89.83%	–	34.02	< 0.01
PV_+_	90.06%^a^	81.42%	–	22.74	< 0.01
PV_−_	92.98%^a^	95.24%	–	6.78	0.01
Consistency	92.07%^a^	90.16%	–	5.06	0.02
YI	80.71%	80.68%	–		
Kappa (95% CI)	0.82 (0.79, 0.85)	0.78 (0.75, 0.81)	–		

*PV*_*+*_ Positive predict value, *PV*_*−*_ Negative predict value, *YI* Youden index

^a^compared with 48 h and 72 h or compared with 48 h

The sensitivity of erythema measurement was 85.33, 90.85, and 91.08% at 24 h, 48 h, and 72 h after EC skin test, respectively; the sensitivity was the lowest at 24 h (χ^2^ = 16.18, *P* < 0.01), and there was no statistical difference between the sensitivity at 48 h and 72 h (χ^2^ = 0.03, *P* = 0.88). The specificity of erythema measurement was 95.38 and 89.83% at 24 h and 48 h after EC skin test, respectively; the specificity was lower at 48 h (χ^2^ = 34.02, *P* < 0.01). Kappa values were 0.82 at 24 h and 0.78 at 48 h. The consistency of 24 h and 48 h after the skin test between erythema and clinical diagnosis were more than 90%; at 48 h after a skin test, the consistency was slightly lower than that at 24 h after skin test (χ^2^ = 5.06, *P* = 0.02). The remaining results are shown in Table 2.

When comparing the consistency between induration with a clinical diagnosis and erythema with clinical diagnosis, there was a significant difference at 24 h after EC skin test (χ^2^ = 11.34, *P* <  0.01), and no difference at 48 h after skin test (χ^2^ = 1.99, *P* = 0.16). The consistency of erythema with the clinical diagnosis was higher than that of induration with clinical diagnosis at 24 h after a skin test, and there was no difference in the consistency between two of them at 48 h after the skin test.

### The comparison of sensitivity and specificity between induration and erythema

In TB patients, there were more patients with positive erythema response than those with positive induration response. The sensitivity in judging induration and erythema were compared at three time points, *P* < 0.01 (χ^2^_24 h_ = 38.60, χ^2^_48 h_ = 14.82, χ^2^_72 h_ = 17.93) (Table 3). These results demonstrated that the sensitivity of erythema measurement was significantly higher than that of induration. In bacteriologically positive TB patients, the sensitivity of erythema measurement was higher than that of induration, *P* < 0.01 (χ^2^_24 h_ = 20.72, χ^2^_48 h_ = 11.16, χ^2^_72 h_ = 16.58) (Table 3). In bacteriologically negative TB patients and extrapulmonary TB patients, there was no differences in sensitivity between induration and erythema measurement at 48 h and 72 h after a skin test, *P* ≥ 0.05 (Table 3).

**Table 3:** The sensitivity and specificity of induration and erythema after EC skin test

	24 h		48 h		72 h	
	≥5 mm, N	<5 mm, N	≥5 mm, N	<5 mm, N	≥5 mm, N	<5 mm, N
In TB patients						
Induration diameter	536	207	626	117	620	120
Erythema diameter	634	109	675	68	674	66
χ^2^	38.60		14.82		17.93	
*P*	<0.01		<0.01		<0.01	
In bacteriologically positive TB patients						
Induration diameter	303	91	341	53	332	62
Erythema diameter	351	43	369	25	368	26
χ^2^	20.72		11.16		16.58	
*P*	<0.01		<0.01		<0.01	
In bacteriologically nagative TB patients						
Induration diameter	188	94	227	55	231	48
Erythema diameter	227	55	244	38	244	35
χ^2^	13.87		3.72		2.39	
*P*	<0.01		0.05		0.12	
In extrapulmonary TB patients						
Induration diameter	46	21	58	9	57	10
Erythema diameter	56	11	62	5	62	5
χ^2^	4.11		1.28		1.88	
*P*	0.04		0.26		0.17	
In healthy volunteers						
Induration diameter	38	1476	134	1380	–	–
Erythema diameter	70	1444	154	1360	–	–
χ^2^	9.83		1.54			
*P*	<0.01		0.22			
In BCG vaccination volunteers						
Induration diameter	20	295	16	299	–	–
Erythema diameter	29	286	22	293	–	–
χ^2^	1.79		1.01			
*P*	0.18		0.32			

Among healthy volunteers, there were more subjects with negative induration response than those with negative erythema response. The specificity for analyzing induration and erythema were compared. There was a significant difference at 24 h after EC skin test (χ^2^ = 9.83, *P* < 0.01), and no difference at 48 h after EC skin test (χ^2^ = 1.54, *P* = 0.22) (Table 3), which suggested that the specificity of erythema measurement was lower than induration at 24 h after skin test, but not at 48 h after skin test.

In BCG vaccination volunteers, the specificity of induration and erythema measurement were > 90%, and the specificity were compared at two time points, *P* > 0.05 (χ^2^_24 h_ = 1.79, χ^2^_48 h_ = 1.01). These proved there was no difference in the specificity between induration and erythema (Table 3).

### Comparison the consistency of induration and erythema

In TB patients, the CR_+_ (χ^2^ = 31.99, *P* <0.01), Kappa value, and consistency (χ^2^ = 18.36, *P* < 0.01) between induration and erythema were lower at 24 h after the skin test than that at 48 h and 72 h. In bacteriologically positive TB patients, the CR_+_ (χ^2^ = 8.62, *P* = 0.01) and consistency (χ^2^ = 8.19, *P* = 0.02) between induration and erythema were lower at 24 h after the skin test, while Kappa value was higher at 48 h after skin test. In bacteriologically nagative TB patients, CR_+_ (χ^2^ = 27.02, *P* <0.01), Kappa value and consistency (χ^2^ = 27.27, *P* < 0.01) were lower at 24 h after the skin test. In extrapulmonary TB patients, Kappa value was higher at 48 h after skin test. In healthy volunteers, CR_+_ (χ^2^ = 30.84, *P* < 0.01) and Kappa value at 24 h after skin test were lower than that at 48 h after skin test. The comparisons results are shown in Table 4.

**Table 4:** The Consistency comparison between induration and erythema after EC skin test

	24 h		48 h		72 h		χ^2^	*P*
	Erythema ≥5 mm	Erythema<5 mm	Erythema ≥5 mm	Erythema<5 mm	Erythema ≥5 mm	Erythema<5 mm		
In TB patients								
Induration ≥5 mm	530	6	623	3	618	2		
Induration<5 mm	104	103	52	65	56	64		
CR_+_	83.60%^a^		92.30%		91.69%		31.99	<0.01
CR_−_	94.50%		95.59%		96.97%		0.59	0.75
Consistency	85.20%^a^		92.60%		92.16%		18.36	<0.01
Kappa(95%CI)	0.57 (0.50,0.64)		0.66(0.58,0.74)		0.65(0.57,0.73)			
In bacteriologically positive TB patients								
Induration ≥5 mm	300	3	340	1	331	1		
Induration<5 mm	51	40	29	24	37	25		
CR_+_	85.47%^a^		92.14%		89.95%		8.62	0.01
CR_−_	93.02%		96.00%		96.15%		0.43	0.81
Consistency	86.29%^a^		92.39%		90.36%		8.19	0.02
Kappa(95%CI)	0.53 (0.64, 0.42)		0.58 (0.71, 0.45)		0.52 (0.65, 0.39)			
In bacteriologically negative TB patients								
Induration ≥5 mm	184	4	226	1	231	0		
Induration<5 mm	43	51	18	37	13	35		
CR_+_	81.06%^a^		92.62%		94.67%		27.02	<0.01
CR_−_	92.73%		97.37%		100.00%		3.25	0.20
Consistency	83.33%^a^		93.26%		95.34%		27.27	<0.01
Kappa(95%CI)	0.58 (0.68, 0.48)		0.76 (0.86, 0.66)		0.82 (0.92, 0.72)			
In extrapulmonary TB patients								
Induration ≥5 mm	45	1	58	0	57	0		
Induration<5 mm	11	10	4	5	5	5		
CR_+_	80.36%^a^		93.55%		91.94%		6.07	0.05
CR_−_	90.91%		100.00%		100.00%		0.96	0.62
Consistency	82.09%^a^		94.03		92.54%		6.06	0.05
Kappa(95%CI)	0.52 (0.30, 0.74)		0.68 (0.40, 0.97)		0.63 (0.34, 0.92)			
In healthy volunteers								
Induration ≥5 mm	35	3	131	3	–	–		
Induration<5 mm	35	1441	23	1357	–	–		
CR_+_	50.00%^a^		85.06%		–	–	30.84	<0.01
CR_−_	99.79%		99.78%		–	–	0.01	0.94
Consistency	97.49%		98.28%		–	–	2.30	0.13
Kappa(95%CI)	0.64(0.53,0.75)		0.90(0.86,0.94)		–	–		

*CR*_*+*_ Positive coincidence rate, *CR*_*−*_ Negative coincidence rate

^a^compared with 48 h and 72 h or compared with 48 h

Above all, induration and erythema could be used for *M. tuberculosis* detection. These data suggested that the best sensitivity, specificity, and other diagnostic results are obtained 48 h after the skin test is performed.

### Comparison of diagnostic indicators for the EC skin test

We selected induration, erythema, induration or erythema, induration and erythema as the diagnostic indicators of the EC skin test, and compared the diagnostic effects of these four indicators in different types of patients at 48 h after EC skin test .

In different types of patients, the sensitivity of induration or erythema measurement was the highest, the sensitivity of induration and erythema measurement was the lowest, however, in extrapulmonary TB patients, there were no differences in the sensitivity of the four indicators. The specificity of induration or erythema measurement was the lowest, the specificity of induration and erythema measurement was the highest, however, there was no difference in the specificity of the four indicators. In different subjects, there was no difference in the consistency of the four indicators with clinical diagnosis. Detailed data are shown in Table 5.

**Table 5:** Comparison of the four diagnostic indicators for the EC skin test

	TB patients VS. Healthy volunteers	Bacteriologically positive TB patients VS. Healthy volunteers	Bacteriologically negative TB patients VS. Healthy volunteers	Extrapulmonary TB patients VS. Healthy volunteers
Sensitivity				
Induration	84.25%	86.55%	80.50%	86.57%
Erythema	90.85%^a^	93.65%^a^	86.52%^a^	92.54%
Induration or Erythema	91.25%^a^	94.16%^a^	86.88%^a^	92.54%
Induration + Erythema	83.85%	86.29%	80.14%	86.57%
χ^2^	33.50	24.98	8.37	2.55
*P*	<0.01	<0.01	0.04	0.47
Specificity				
Induration	91.15%	91.15%	91.15%	91.15%
Erythema	89.83%	89.83%	89.83%	89.83%
Induration or Erythema	89.63%	89.63%	89.63%	89.63%
Induration + Erythema	91.35%	91.35%	91.35%	91.35%
χ^2^	4.13	4.13	4.13	4.13
*P*	0.25	0.25	0.25	0.25
Consistency				
Induration	88.88%	90.20%	89.48%	90.96%
Erythema	90.16%	90.62%	89.31%	89.94%
Induration or Erythema	90.16%	90.57%	89.20%	89.75%
Induration + Erythema	88.88%	90.30%	89.59%	91.14%
χ^2^	3.97	0.27	0.17	2.71
*P*	0.26	0.97	0.98	0.44

^a^compared with Induration or Induration + Erythema

## Discussion

Currently, there is no golden standard for LTBI diagnosis. Therefore, in order to detect *M. tuberculosis* infection, TB patients were used instead of LTBI [28–30]. All enrolled patients underwent a bacteriological test of *M. tuberculosis*. Based on the bacteriological results, imaging results, and clinical symptoms, patients were divided into bacteriologically positive and negative patients, and extrapulmonary tuberculosis patients. In previous clinical trials, we validate the consistency and safety of the recombinant fusion protein EC for detecting *M. tuberculosis*. In this phase III clinical trial, we expanded the number of subjects and found that induration or erythema diameter not less than 5 mm could be used as a diagnostic indicator for detecting *M. tuberculosis* infection.

Combined with the results of previous clinical trials [31, 32], these data suggested that the best sensitivity, specificity, and other diagnostic results are obtained 48 h after testing. Furthermore, we found that diagnostic results of induration and erythema had higher sensitivity, specificity, consistency, and Kappa values compared with clinical diagnosis. Therefore, we believed that induration and erythema could be used as diagnostic indicators for EC skin test to detect *M. tuberculosis* infection. On the one hand, the recombinant fusion protein is more stable than recombinant mixed protein [33] and could produce a stronger cellular immune response. Thus, the erythema response could not be ignored. On the other hand, EC skin test reagent contains only *M. tuberculosis-*specific antigens proteins; skin responses should be specific. Therefore, induration and erythema as skin response should be measured.

Previous small sample clinical trial suggested that erythema was a specific response to the EC skin test. After the test, the sensitivity of the erythema was higher then 80%, the specificity was higher then 90%, and the test results were consistent with clinical diagnosis results (Kappa value ≥0.78). In the BCG vaccination population, more than 90% of the subjects had a negative erythema response. A large prospective study of PPD skin test conducted in Japan confirmed that the specificity of erythema measurement was closely related to induration, and erythema was more likely to produce a specific response without being confused by induration [34]. Moreover, another study showed that erythema and induration had the same response trend. In this study, subjects were divided into *M. tuberculosis* infection negative and positive groups according to the erythema response [35]. We believed that erythema should be a specific response to the EC skin test according to the above results. Therefore, we included erythema as a judgement indicator and further verified its detection effect by expanding the sample size. In TB patients, the sensitivity of erythema measurement was higher than that of induration. If only the induration is used, many patients may be misdiagnosed, which could reduce the sensitivity of the test. In this study, erythema could improve the sensitivity of the test in TB patients. Although the erythema response had a certain false-positive rate in healthy volunteers, the false positive rate was low, and its specificity was still high. Especially in BCG vaccination volunteers, whether induration or erythema, its specificity was higher than 90%, which explained that EC skin test response would not be affected by BCG. In addition, the analysis found that the consistency of erythema and clinical diagnosis was high. Overall, the erythema response in different types of TB patients kept the sensitivity of about 90% after 48 h; its sensitivity was higher than that of induration. In healthy volunteers, the erythema had a specificity of about 90%. Above all, the sensitivity and specificity of the erythema response were both high. Erythema used as the judgment indicator can improve the positive detection rate in TB patients without affecting the specificity of healthy volunteers. Therefore, erythema was a specific response to the EC skin test and could be used for detecting *M. tuberculosis* infection. We believed that erythema could be used as a diagnostic indicator of the EC skin test.

The skin test response produced in subjects showed that the diameter of erythema was larger, smaller, or equal to the diameter of induration. Therefore, it was one-sided and unreasonable if uniformly stipulated that it used induration alone as the diagnostic indicator for the EC skin test. The CR_+_ and the consistency of induration and erythema were higher than 90%, and the Kappa value was higher than 0.66 in different types of TB patients at 48 h after testing, which indicated the consistency of induration and erythema results was better in patients. The CR_−_ of induration and erythema was more than 99% in healthy volunteers. Notably, the consistency was 98.28%, and Kappa value was 0.90 at 48 h after testing, indicating that the consistency of induration and erythema results was excellent in healthy volunteers. Since induration and erythema had a good consistency, both of them could be used as diagnostic indicators for EC skin test.

In terms of sensitivity, the use of induration and erythema in parallel was higher than that when the indicators used alone or in tandemly; In terms of specificity, although the two indicators used in parallel were lower than the two indicators used alone or in tandemly, there was no statistical difference. In terms of consistency with clinical diagnosis, the consistency of the four diagnostic indicators was about 90%. In summary, we believed that the parallel combination of induration and erythema should be used to evaluate the responses of the EC skin test.

In this study, only yellow-skinned people were included. People with darker skin will have a specific effect on the interpretation of erythema results. Therefore, in the darker-skinned population, we mainly take the diagnosis of induration, supplemented by the diagnosis of erythema.

The above content was only part of the phase III clinical trial for the EC skin test. In phase III clinical trials, we performed a PPD skin test, EC skin test, and T-SPOT test in TB patients and healthy volunteers, respectively. Our study indicated that an EC skin test can be used for screening *M. tuberculosis* infection and was an effective skin test reagent for clinical auxiliary diagnosis of tuberculosis. No adverse events and serious adverse events leading to withdrawal occurred in a clinical trial. In terms of sensitivity, the EC skin test was equivalent to the PPD skin test and T-SPOT test. In terms of specificity, the EC skin test was superior to the PPD skin test and non-inferior to the T-SPOT test. Compared the consistency of the three tests and clinical diagnosis, it was concluded that the consistency of PPD skin test and clinical diagnosis was lower than that of EC skin test or T-SPOT and clinical diagnosis.

## Conclusion

Induration and erythema could be used as diagnostic indicators for EC skin test. Erythema as diagnostic indicators could improve the sensitivity of the EC skin test, without affecting the specificity. Induration or erythema diameter not less than 5 mm could be as a diagnostic indicator for positive response of the EC skin test. Although the determination of skin test results is subjective, the EC skin test is more specific than TST, and can be used to detect *M. tuberculosis* infection early, quickly, and accurately. Due to its convenient operation, it is more suitable to be used in countries with high TB burden to reduce the financial burden.

## Supplementary information

**Additional file 1.** Diagnostic criteria for pulmonary tuberculosis (WS288-2008).

## Data Availability

The datasets used and/or analysed during the current study are de-identified and available from the corresponding author on reasonable request.

## Abbreviations

TB: Tuberculosis
M. tuberculosis: *Mycobacterium tuberculosis*
LTBI: Latent tuberculosis infection
PPD: Pure protein derivative
IFN-γ: Interferon-γ
TST: Tuberculin skin test
IGRAs: Interferon-γ release assays
NTM: Non-tuberculous mycobacteria
BCG: Bacillus Calmette-Guérin
ESAT6: 6 kDa early secretory antigenic target
CFP10: 10 kDa culture filtrate protein
EC: ESAT6-CFP10
PV_+_: Positive predict value
PV_−_: Negative predict value
YI: Youden index
CR_+_: Positive coincidence rate
CR_−_: Negative coincidence rate
