# Supplementary material for: Induration or erythema diameter not less than 5 mm as results of recombinant fusion protein ESAT6-CFP10 skin test for detecting M. tuberculosis infection
Source: BMC Infect Dis. 2020 Sep 18;20:685. doi: 10.1186/s12879-020-05413-9 (PMC7501602; doi:10.1186/s12879-020-05413-9)
Supplement: Supplementary file 1 — Additional file 1. Diagnostic criteria for pulmonary tuberculosis (WS288-2008). [file 12879_2020_5413_MOESM1_ESM.doc]

**Diagnostic criteria for pulmonary tuberculosis (WS288-2008)**

A definite diagnosis of pulmonary tuberculosis can be made according to the following criteria.

1. **Diagnosis of sputum smear-positive pulmonary tuberculosis**

At least three sputum samples of patients are collected. Smears are observed under a microscope, and 300 fields per sample are examined for acid-fast bacilli. Culture examination for *Mycobacterium tuberculosis* should been conducted if possible. Patients who meet one of the following criteria are diagnosed as sputum smear-positive pulmonary tuberculosis:

1. acid-fast bacillus smear-positive in two sputum smear samples;
2. acid-fast bacillus smear-positive in one sputum smear samples, and positive pathological findings on chest X ray (such as upper-lobe infiltrates, cavity in-filtrates and/or hilar or paratracheal adenopathy).
3. acid-fast bacillus smear-positive in one sputum smear samples, and positive culture for *Mycobacterium tuberculosis* in one sputum sample.
4. **Diagnosis of sputum** **culture positive pulmonary tuberculosis**

Patients who have positive pathological findings on chest X ray, acid-fast bacillus sputum smear-negative, and positive culture for *Mycobacterium tuberculosis* are diagnosed as sputum culture positive pulmonary tuberculosis.

1. **Pathological diagnosis of pulmonary tuberculosis**
